# Psychosocial Factors Explaining Academic Dropout among Scholarship Students at a Public University in Peru

**DOI:** 10.12688/f1000research.174541.2

**Published:** 2026-08-21

**Authors:** Yefferson Llonto-Caicedo, Guido Alarcón-Villanueva, Ofrmar Dionell Jiménez-Garay, Debora Margarita De Jesus Paredes-Olano, Yessica Marilu Acuña-Romero, Isaí Geremias Ruiz-Díaz, Diana Marilú Sánchez-Alamo, Jorge Luis Vargas-Espinoza, Oscar Jovani Zambrano-Infante, Rogger Orlando Morán-Santamaría

**Affiliations:** 1 Lambayeque, Universidad Nacional Pedro Ruiz Gallo Facultad de Ciencias Economicas Administrativas y Contables, Lambayeque, Lambayeque, Peru; 2 Ica, Universidad Nacional San Luis Gonzaga, Ica, Ica, Peru; 3 Lima, Universidad Tecnologica del Peru, Lima, Lima, Peru; 4 Cajamarca, Universidad Nacional de Cajamarca, Cajamarca, Cajamarca, Peru; 5 Amazonas, Universidad Nacional Intercultural Fabiola Salazar Leguia de Bagua, Bagua, Amazonas, Peru; 6 San Martin, Universidad Nacional de San Martin, Tarapoto, San Martin, Peru

**Keywords:** Student dropout; Higher education retention; Psychosocial factors; Scholarship students; Academic integration; Social integration; Structural equation modeling (SEM); PLS-SEM; Peru.

## Abstract

**Introduction:**

Student dropout in higher education is one of the most concerning issues on the agendas of countries, and it remains a challenge for the higher education system. This is reflected in cases where, despite the benefits and support provided by the state, students decide to give up their scholarships for various reasons. These reasons include economic, social, psychological, organizational, and interaction-related factors, as well as internal and external factors, all of which highlight the inefficiency of the higher education system.

**Objective:**

To examine the associations of social and psychological factors with academic and social integration among scholarship students with low academic performance at a Peruvian public university in 2024.

**Methods:**

The methodology involves a quantitative approach, with a descriptive-explanatory level and a non-experimental design, as it is framed within the social sciences.

**Results:**

The results of the structural equations rely on the confirmatory factor analysis, whose indices yield the following outcomes: χ ^2^ = CMIN/df = 1.649, GFI = 0.923, AGFI = 0.955, NFI = 0.906, CFI = 0.996, RMSEA = 0.081 and SRMR = 0.100. These values demonstrate adequate indices for the constructs in the estimated model, which is explained by the six factors associated with academic and social integration in the analyzed sample.

**Conclusions:**

The social factors that students consider relevant to academic dropout relate to issues such as difficulties in making friends, the institution failing to provide conditions for healthy social interaction, and family environment. Meanwhile, the motivational factors that students consider relevant to academic dropout pertain to issues related to emotional aspects that influence decision-making, the normative aspect in terms of understanding how higher education functions, and psychological health.

## 1. Introduction

It is evident that higher education plays a pivotal role in the competitive and sustainable development of a nation. This assertion is corroborated by research findings that demonstrate a positive correlation between university education and higher economic income, in comparison to those who do not attain this level of education. ^1^ The impact of this phenomenon is twofold: it is beneficial to the individual, and it is also conducive to the development of a skilled workforce that is able to drive innovation and productivity. Nevertheless, the efficacy of the system is undermined when educational institutions are unable to maintain a high retention rate of their students. This indicates a discrepancy between the system's design and the actual requirements of the population. ^2– 4^

In order to optimise their potential, it is essential that systems guarantee not only access but also retention, by integrating mechanisms that identify and mitigate early barriers. The economic return on higher education has encouraged its promotion as an engine of competitiveness. However, this process faces a critical challenge in the limited functionality of education systems in retaining students. ^5, 6^

This problem is indicative of a multifaceted nature, manifesting as a consequence of numerous underlying factors. These include, but are not limited to, inadequate curricular adaptation and an absence of sufficient consideration for psychosocial elements such as academic stress and identity disconnection. These elements are further compounded in contexts characterised by inequality, where institutional entities perpetuate exclusionary dynamics rather than addressing them. This phenomenon is indicative of the inefficiencies inherent in systems that prioritise access over retention, without addressing the underlying causes, such as a lack of prior academic preparation or the absence of emotional support networks. First-generation university scholarship recipients, in particular, encounter a “culture shock” when entering academic environments that fail to consider their sociocultural backgrounds. This can result in feelings of isolation and stress. Furthermore, the presence of institutional bureaucracy, manifesting in the form of protracted processes for the resolution of administrative issues, has been demonstrated to intensify feelings of frustration and result in elevated rates of attrition. This issue has been identified as a priority challenge for global agendas, as demonstrated by the case of students who, despite being recipients of state scholarships, discontinue their studies due to economic, psychological or institutional factors. ^7, 8^

In Peru, the proportion of young people who have access to higher education is only 30%, which is lower than the proportion in countries such as Chile and Colombia. ^9^ This disparity is further accentuated when analysing expenditure per individual: while two out of ten young people in the lowest quintile manage to enrol, this figure rises to five out of ten in the highest quintile. The geographical concentration of educational resources in the capital, Lima, has been identified as a contributing factor to the exacerbation of the educational disparities between regions. A significant proportion of university enrolment is concentrated in Lima, with 41% of all enrolments being located within the city. This has the effect of limiting access to educational opportunities in regions that may not possess the same level of educational infrastructure as Lima. This phenomenon of territorial inequality is indicative of systems that demonstrate a bias towards urban and higher-income populations, thereby marginalising those inhabiting rural or peri-urban areas. Furthermore, the absence of public universities in the provinces compels a significant number of students to migrate, resulting in expenses that, when combined with job insecurity, heighten the likelihood of attrition. It is evident that, in the absence of effective decentralisation and regional inclusion policies, the system is likely to continue exhibiting exclusionary tendencies.

Conversely, 65% of the university-age population encounter economic impediments to accessing higher education, a phenomenon that has given rise to policies such as scholarships and educational loans. ^10– 12^ Nevertheless, these mechanisms frequently prove to be inadequate when not accompanied by academic and psychosocial support. Consequently, scholarship recipients from public educational institutions may encounter deficiencies in their prior learning, which can result in their falling behind and, ultimately, dropping out of their studies. Furthermore, the paucity of information regarding the requirements for maintaining a scholarship, such as minimum grade point averages, has been shown to engender feelings of anxiety and demotivation among scholarship recipients.

Consequently, the phenomenon of university dropout can be considered as multi-causal, as posited by, ^13^ thus necessitating interdisciplinary approaches to facilitate comprehension. From a psychological standpoint, factors such as academic self-concept, resilience to failure, and disconnection from the educational project have been demonstrated to influence the decision to discontinue one's education. ^14^ Conversely, the sociological approach to understanding the phenomenon of dropout, as articulated in the seminal work of, ^15^ undertakes a multifaceted examination of salient variables including family income, parental educational attainment, and gender.

A comprehensive understanding of the phenomenon of scholarship students dropping out of higher education necessitates an examination of both socioeconomic and psychological factors. As asserted by Refs. ( 16 and 17), economic precariousness imposes limitations on both access and retention. To illustrate this point, consider a scholarship student who is obliged to contribute financially to their family. In such a scenario, they may find themselves compelled to prioritise securing informal employment over their academic pursuits. From a psychological standpoint, the pressure to maintain high grades in order to retain scholarship funding has been shown to engender chronic stress, which in turn has a detrimental effect on performance. The interaction of these factors within education systems that lack safety nets, such as supplementary subsidies or academic flexibility, results in scholarships serving merely as temporary remedies rather than comprehensive solutions. In order to break this cycle, there is a necessity for policies that recognise the multiple dimensions of vulnerability. Such policies must integrate financial, academic and emotional support in a synergistic manner.

The Peruvian university sector is currently undergoing a series of reforms to its National Policy on Higher University Education. A key element of these reforms is the identification of the most pertinent factors to inform the development of strategies to address the issue of increasingly restricted access to university life. This assertion is supported by empirical evidence derived from rigorous research. The present study seeks to explore the psychosocial factors that explain academic dropout among scholarship students at a public university in 2024. The primary motivation underlying this research endeavour is to make a substantial scientific and practical contribution to policy makers in the domain of strategy design.

Academic performance and dropout are related educational outcomes, but they are conceptually distinct. Low grades, failed courses, and limited curricular progress are indicators of vulnerability or predictors of dropout; however, they do not by themselves demonstrate that a student has permanently discontinued their studies. Dropout requires a definition based on verifiable behavior, such as institutional withdrawal, failure to re-enroll for a specified period, or departure from the higher education system. Accordingly, in this study, an academic average below 10 was used exclusively as a criterion to identify academically vulnerable students at potential risk of dropout. The analysis focused on examining the association of social and psychological factors with these students’ academic and social integration. ^18, 19^

## 2. Theoretical framework

### 2.1 Models of university dropout

According to Himmel, ^20^ the term ‘university dropout’ is defined as the severing of ties with academic enrolment. This can be caused by various factors, such as financial difficulties, problems adapting to the university environment, or lack of motivation. This period is distinguished by the premature termination of university studies, marked by an extended absence of the student. ^21^ For Ref. 22, the term ‘dropout’ is employed to denote either the voluntary or involuntary termination of enrolment in an academic programme. The former is characterised by the individual's own decision to disengage from their studies, while the latter is precipitated by substandard academic performance. The phenomenon of 'dropout' thus engenders the act of withdrawal. The interruption may be temporary or permanent, voluntary or forced, and manifests itself in different ways, as well as the multi-causal problem presented by the diversity of students who are admitted to the university system. ^23^ As asserted by Ref. Barroso et al., ^24^ the dropout process can be subdivided into two critical periods. Firstly, there is the initial contact with the institution, and secondly, there is the period during the first semesters of the programme. This is due to the fact that the social adaptation programme and the academic aspect begin when students come into contact with the university environment. The relevant study Ref. Tinto ^23^ provides a social perspective on university dropout, emphasising the significance of students' social integration. Social integration can be defined as the process by which students establish meaningful relationships with peers, professors and the institution itself. The hypothesis that students who feel integrated are more likely to persist in their studies is one that merits further investigation. Conversely, a paucity of social integration can engender feelings of isolation and frustration, which in turn can increase the risk of dropout.

Social integration in the university environment is defined as the capacity of students to establish meaningful connections with their peers, teaching staff, and the institution itself. This process is of fundamental importance in the context of academic retention, insofar as it engenders a sense of belonging and motivation, thereby reinforcing commitment to studies. Recent research has indicated that social integration is a pivotal factor in student retention, and its absence can considerably increase the likelihood of university dropout. ^25^ As asserted by Tlalajoe-Mokhatla, ^25^ the prevailing paradigms of retention have accorded precedence to the pursuit of academic integration, while neglecting to address the necessity of social integration. However, it is imperative to recognise that the simultaneous consideration of both dimensions is indispensable for the enhancement of graduation rates. The present author hypothesises that social learning and the implementation of intentional support strategies have the capacity to reduce rates of student attrition and enhance the student experience in higher education. Conversely, a paucity of social integration can engender a sense of alienation, which may, in turn, precipitate a decline in motivation and ultimately result in academic withdrawal. A study undertaken at the University of Granada discovered that students who withdrew from their studies exhibited marked disparities in domains such as academic and social integration, university stress, and institutional commitment. The findings of the research indicate that individuals with a reduced degree of social integration encounter heightened challenges in coping with the demands of university life, a factor that increases their probability of departing the institution during their early years of study. ^18^

In a comparative analysis, Ref. Franz and Paetsch ^26^ investigated the differences in social integration between teacher training students and those in other university courses. The findings indicated that prospective educators exhibited a propensity to establish stronger connections with their peers, while concurrently cultivating less pronounced relationships with their instructors. This dynamic exerted a significant influence on their decision to persist in their academic pursuits or discontinue their studies. This finding serves to reinforce the importance of interaction with the educational community in university retention. The present study provides a compelling illustration of the impact of social integration on university dropout rates, with particular reference to students who are engaged in paid employment while pursuing their academic studies. Research conducted in Estonia, Lithuania and Poland revealed that employed students who maintained positive relationships with their teachers and peers were less likely to discontinue their studies. However, students encountering academic challenges and a sense of alienation from the university community exhibited higher rates of attrition. This study underscores the significance of university social capital in the context of student retention, as evidenced by the seminal work of. ^27^

In the Latin American context, research conducted in Ecuador has identified a number of factors associated with university dropout, including personal, family and economic factors. However, it was also highlighted that a lack of integration with the university community was a determining factor in the decision to drop out. It has been demonstrated that students who are unable to establish meaningful connections with their academic environment are more likely to leave university. This suggests that retention strategies should focus on strengthening social inclusion within institutions. ^28^

Meanwhile, scientific literature refers to academic productivity and intellectual growth involving interactions with teachers and authorities. These interactions are reflective of beliefs about one's goals, which are affected by both institutional and other external factors. ^29^

### 2.2 Social factors

In the present study Himmel, ^30^ the researcher considered the role of external factors in university academic dropout, in addition to psychological aspects. This was approached through the lens of sociological models. Therefore, it is evident that Durkheim's findings, as outlined in Ref. Gross & Niman, ^29^ contribute to the existing body of knowledge on the subject. Specifically, the study draws upon suicide theory, asserting that the dissolution of both the social system and social affiliation exert a direct influence on the incidence of suicide. In other words, the research considers the comprehensive perspective of students, acknowledging the interplay between the higher education environment and social integration on the decision to discontinue their education. This analysis identifies factors such as students' sense of alienation from their immediate environment and familial influences, which impact both academic and normative aspects of their lives. It is evident that there is a multifaceted relationship between the family environment and intellectual power and articulation from a normative perspective. This normative perspective is predicated on traditional academic performance and the relevance of impacting intellectual power, peers, and integration. This, in turn, has been shown to result in student satisfaction and commitment to the institution, which, as a consequence, leads to the decision to drop out. ^31^

It is therefore evident that social aspects are pertinent in the context of the direct correlation between academic performance and the decision to discontinue formal education. The theoretical model demonstrates the influence of individual student characteristics on the likelihood of attrition, which is a matter of concern both in terms of the economic implications for the state and the role of familial and social environments in shaping academic performance. The investigation of the influence of social mechanisms on student academic performance is therefore of paramount importance. ^32^

Within the domain of social factors, education assumes a pivotal role in the development of social consciousness and the propagation of values and norms to subsequent generations. ^33^ Education is regarded not only as a means of preparing individuals for the world of work, but also as a conduit for the maintenance and transmission of culture and social solidarity. Meanwhile, the Ref. Gobato ^34^ posits that, in consideration of its functionalist approach, sociological theory examines the manner in which social institutions contribute to the well-being and stability of society. Within this paradigm, education is regarded as an institution that fulfils multiple functions, including the transmission of knowledge, the assignment of roles, and the selection and classification of individuals within society. Conversely, it has been contended that educational institutions can engender anomie if they fail to provide all individuals with equal opportunities to achieve socially valued success. As posited by Ref. Vega et al., ^31^ the paucity of equitable access to education has been demonstrated to engender deviance and frustration.

Whilst education can offer opportunities for advancement in the social hierarchy, it was also noted that inequalities in access to quality education can hinder mobility and contribute to the reproduction of social inequality. ^22^ Therefore, from an educational perspective, social structures provide differential opportunities to individuals according to their position within the structure. It has been demonstrated that such opportunity structures can play a pivotal role in the establishment and perpetuation of social inequalities. ^14^

As posited by Pérez et al. ^35^ education is currently experiencing a social crisis, characterised by the challenges confronting educational institutions in the effective transmission of knowledge and values to successive generations. The argument is posited that factors such as the diversification of society, changes in authority and the role of the family contribute to this crisis.

Contemporary society is characterised by its diversity in terms of cultures, values and knowledge. Villegas et al. ^36^ draws attention to the challenge faced by educational institutions in adapting to this diversification and identifying effective methodologies for the transmission of a unified body of knowledge and values that are pertinent and meaningful to all students, irrespective of their cultural and social contexts.

Consequently, educational institutions have ceased to be the exclusive conduits of knowledge, as alternative institutions and media have assumed significant roles in shaping public opinion and facilitating knowledge acquisition. ^22^ This indicates that the crisis of transmission gives rise to the emergence of novel forms of inequality. Individuals with access to supplementary educational resources beyond the formal education system are likely to benefit from a more comprehensive education, while those who rely exclusively on formal schooling may encounter difficulties in acquiring the necessary skills and knowledge. ^13^

From a neo-institutionalist perspective, educational institutions are regarded as normative structures that define the rules of the game in society. It is important to note that these rules encompass not only formal policies and practices, but also cultural and social norms that exert influence on the behaviour of actors within the education system. ^31^

The concept of institutionalisation, as it pertains to the tendency of organisations to emulate the structures and practices of other organisations, is of particular relevance in this context. This phenomenon, known as institutionalisation, is characterised by three distinct mechanisms: coercive, mimetic, and normative pressures. Within the domain of education, this phenomenon may be evidenced by the adoption of analogous policies and practices among educational institutions. ^22^

This approach considers the conditions of the environment, both external and internal, that affect the functioning of educational institutions. This may encompass factors such as competition between educational institutions, shifts in the demand for education, and government policies. ^37^

From a sociological perspective Labraña, ^38^ higher education institutions are conceptualised as entities in a state of constant evolution and adaptation. The process of transformation in higher education is driven by a complex interplay of endogenous and exogenous factors, which, when aligned with the overarching objective of promoting social development, can significantly impact the teaching and learning processes. This perspective, as outlined by Weber, emphasises a specific, structurally social and formalised purpose, where economic and social factors interweave to influence individual behaviour within a social environment. This environment, in turn, responds to imbalances that extend from moral patterns to human action.

### 2.3 Psychological factors

Fishbein and Ajzen ^39^ in their theory of reasoned action, consider a psychological approach that addresses the identification of the real aspects and characteristics of those students who generate a strong intention to achieve their goal. The model under discussion addresses three key factors related to previous behaviours, attitudes and subjective norms from which human behaviour is approached.

The attitudinal aspect involves the analysis of the cognitive, affective and behavioural dimensions. This is achieved by means of an examination of the behaviour exhibited by individuals who tend to adopt positive, negative or neutral attitudes. In the case of the student model, achievement in the attitudinal aspect is influenced by factors related to persistence, selection and academic performance, which implies the attitude towards the intellectual aspect. ^40^

The behavioural aspect is associated with students' behaviour in relation to their intellectual aspects. This is demonstrated by their behaviour after investing time and effort in obtaining a grant or scholarship, and their behaviour when interacting with the university environment. ^40^

Subjective norms are defined as the normative beliefs that are explained by the perception of pressure towards behavioural intentions, which in turn allow for practical decisions to be made. ^41^

The contemporary era of psychological research has witnessed a paradigm shift towards a more comprehensive understanding of behavioural patterns in relation to students' academic performance. This paradigm shift has been precipitated by the recognition that psychological factors, encompassing cognitive, affective and behavioural dimensions, exert a significant influence on students' behavioural patterns. Consequently, this theoretical contribution posits that human behaviour, in its multifaceted nature, exerts a profound impact on academic performance, which, in turn, affects practical decision-making processes. ^35^

### 2.4 Specification and assessment of the reflective measurement model

The specification of the proposed model is grounded in the theoretical relationship between the construct and its indicators. In reflective models, the construct represents an underlying latent variable that gives rise to the observed responses; therefore, the indicators function as manifestations of the attribute, are expected to covary, and share similar antecedents and consequences. In contrast, in formative models, the indicators represent components that produce or define the construct and therefore are not necessarily interchangeable or expected to be correlated. Model misspecification may distort parameter estimates and lead to incorrect conclusions regarding the structural relationships ^42, 43^

Based on these criteria, the previously described social factors, psychological factors, academic integration, and social integration were conceptualized as reflective constructs because they represent latent attributes manifested through students’ perceptions and responses. Accordingly, the theoretical direction of measurement was specified from each construct to its respective indicators. Before estimation, the conceptual correspondence and polarity of the items were examined to ensure that all items followed the same interpretive direction. Indicators describing antecedent conditions rather than manifestations of the construct were reviewed to avoid a specification incompatible with the reflective nature of the variables.

Accordingly, the social factors, psychological factors, academic integration, and social integration constructs were specified using reflective measurement models, because the indicators were defined as observable manifestations of underlying latent variables. The estimation therefore assumes a causal direction from the constructs to the indicators. Before analysis, items worded in the opposite direction were recoded to ensure a consistent interpretation of the scores.

## 3. Materials and methods

### 3.1 Methodological design

The present study adopts a quantitative approach, thus enabling the phenomenon of academic dropout to be addressed through a descriptive-explanatory design, as has been previously suggested by. ^44^ The methodological choice is substantiated by its capacity to analyse causal relationships and statistical patterns in large samples, thereby facilitating the objective identification of psychosocial factors associated with dropout. At the descriptive level, the characterisation of variables is facilitated by univariate statistics (frequencies), which provide a contextual framework for understanding the profile of the population under study. At the explanatory level, inferential techniques are employed to test hypotheses and ascertain the significant influence of specific factors. This contributes to the study's empirical rigour through structural equations. ^45– 47^

The data collection technique employed was a survey, utilising a structured questionnaire as the primary instrument. This instrument was designed on a Likert scale and administered via a Google Form link for data collection. The survey was disseminated via WhatsApp to a cohort of students exhibiting substandard performance over a two-week period from 14 to 30 July 2024, with an average completion time of 25 minutes.

Ethical considerations and consent in this research consisted of a non-interventional, minimal-risk, anonymous survey administered to adult participants through an online Google Form. The work originated as a formative activity within a postgraduate research methods course in the Master’s programme at the Graduate School of the National University Pedro Ruiz Gallo (UNPRG), and it was not submitted as a thesis- or degree-related protocol; therefore, no formal ethics committee/Institutional Review Board (IRB) approval, waiver, or reference number is available. All procedures adhered to the principles of the Declaration of Helsinki. Participation was voluntary and anonymous; no sensitive or directly identifiable personal data were collected. Electronic informed consent (e-consent) was obtained prior to participation via the first page of the Google Form; only participants who indicated agreement could proceed to the questionnaire. Participants were informed about the study objectives, procedures (approximately 25 minutes), the academic/scientific use of the information, confidentiality safeguards (coding of responses), and their right to withdraw at any time without consequences.

### 3.2 Data collection, population, sample and selection criteria

The population comprised 135 students enrolled in the Beca 18 scholarship program at a Peruvian public university in 2024 who had an academic average below 10. This criterion was used to delimit an academically vulnerable group and served as a proxy indicator of potential university dropout. The study did not include longitudinal records of withdrawal or re-enrollment that would allow confirmed dropout cases to be identified. ^48– 50^

The subject of the research is the beneficiary of Scholarship 18 at a Peruvian public university in the period 2024 who has either dropped out of school or has demonstrated low academic performance.

The procedure used for sample selection is based on the inclusion criterion of having been a scholarship recipient and having a grade point average below 10. This parameter is used for probabilistic sampling to select a sample of 100 respondents, considering a margin of error = 5%, p = 50% and $Z_{\alpha }$ = 95%; calculated to ensure adequate representativeness of the scholarship recipient population.

### 3.3 Data analysis

The social factors, psychological factors, academic integration, and social integration constructs were specified using reflective measurement models because the indicators were defined as observable manifestations of underlying latent variables. Before analysis, items worded in the opposite direction were recoded to ensure that scores had a consistent interpretation.

Given the cross-sectional design, the sample size, and the absence of a direct measure of dropout, the model was used to analyze associations between social and psychological factors and academic and social integration. The measurement model was evaluated before the structural relationships were examined. First, the outer loadings and their significance were assessed through bootstrapping with subsamples. Loadings equal to or greater than 0.708 were considered satisfactory. Indicators with loadings between 0.40 and 0.708 were evaluated by jointly considering their contribution to composite reliability, AVE, and content validity, whereas loadings below 0.40 prompted a substantive review of the indicator.

The data are processed in a scheme that begins by identifying the psychosocial factors that have influenced academic dropout. For the quantitative analysis, the advanced statistical technique of structural equation modelling (SEM) was applied to analyse and model complex relationships between observed and latent variables. its main utility lies in its ability to represent causal and structural relationships through a combination of factor analysis and multiple regression. ^44^

**Figure 1.**
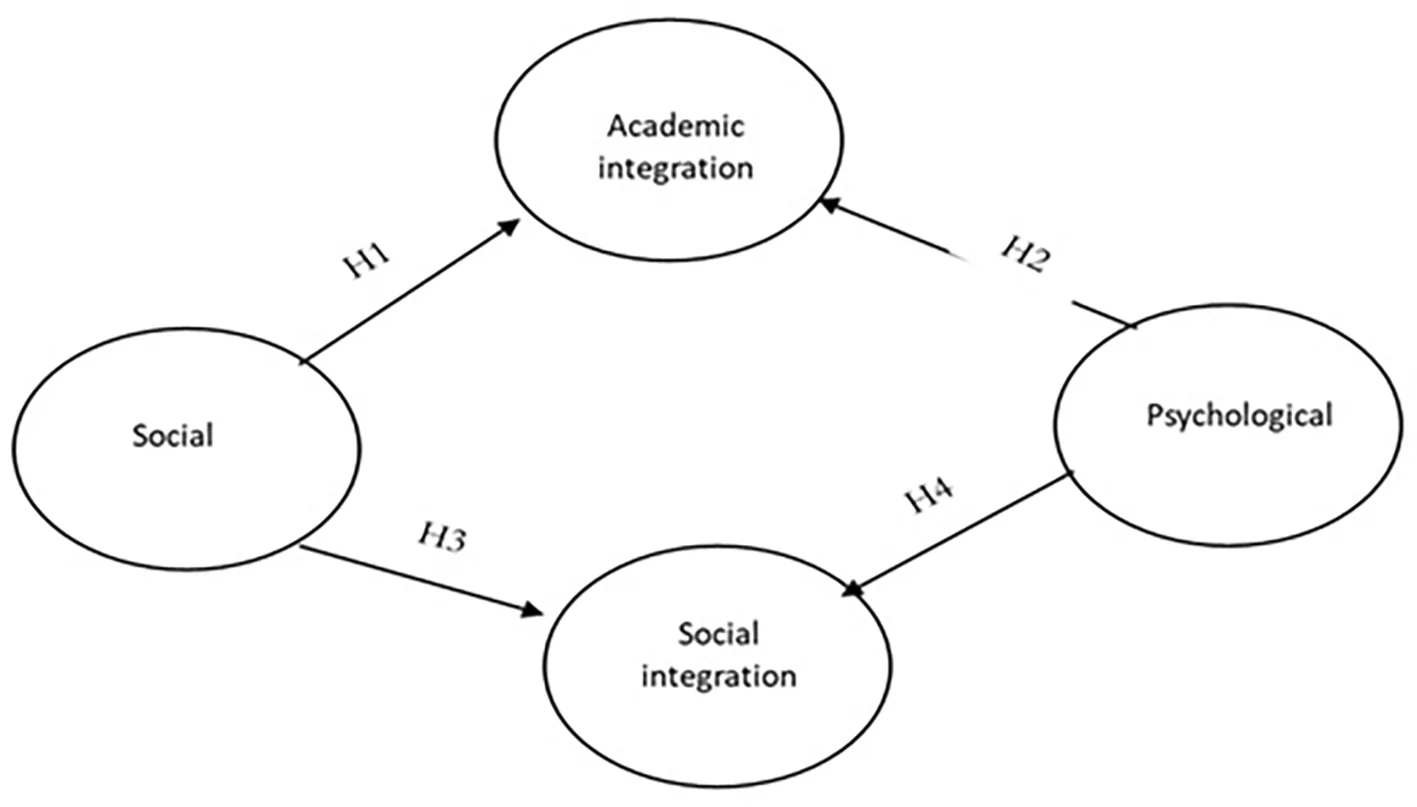
Conceptual model. *Note:* Theoretical design obtained from university dropout models that include social and psychological factors.

On the other hand, the latent variables observed in the data are processed digitally using Microsoft Excel and the specialised programme for structural equation modelling (SEM) data processing, SmartPLS, to analyse the four constructs related to social, psychological, academic integration, and social integration, where 30 observed variables are considered, as presented in Table 1, including the construct shown in Figure 1.

**Table 1.** Observed variables.

Characteristics of respondents	Description	
**Gender**		(1) Male, (2) Female
**Vocational school**		Details of the vocational school
**Variables**	**Description**	
Social	SO1: Integration with the environment	SO11: Finds it easy to make friends in the university environment
		SO12: You integrate easily into any group in your university environment
		SO13: People in the environment tend to support each other
		SO14: The institution is concerned with promoting healthy coexistence in the environment
	SO2: Family environment	SO21: Your family environment has experienced serious difficulties
		SO22: Your family members influence your decisions
		SO23: In your family, household tasks are shared
Psychological	PS1: Previous behaviours	PS11: You have a proactive attitude towards your university studies
		PS12: You are motivated to continue your university career
	PS2: Attitudes	PS21: Always has a positive attitude towards their studies
		PS22: Emotional skills significantly influence their decisions
		PS23: Considers their psychological health to be in optimal condition
		PS24: They attend workshops on managing psychological aspects related to stress
		PS25: Considers that continuing a university career is important for their future life
	PS3: Subjective norms	PS31: Knows the importance of pursuing higher education
		PS32: Is aware of the regulatory aspects of higher education
Academic integration	IA1: University characteristics	IA11: Experiences learning difficulties because the higher education institution faces problems in applying teaching strategies
		IA12: Does not have an organisational role in their studies
		IA13: Perceives the university environment as more difficult than the school environment
	IA2: Institutional characteristics	IA21: Considers university lecturers to be more demanding than school teachers
		IA22: Has inflexible schedules, unlike those at the educational institution
		IA23: Has had problems with the documentation requested by the university
		IA24: Perceives that teachers have not met expectations in teaching their classes
Social integration	IS1: Family characteristics	IS11: Their family has experienced financial problems
		IS12: Has had health problems
		IS13: A family member has had health problems
	IS2: Individual characteristics	IS21: You have some difficulties living alone
		IS22: The amount of time spent with each scholarship recipient or applicant is considered optimal
		IS23: Not entirely sure about the chosen career
		IS24: There is some discrimination due to their habits and/or customs

*Note:* Obtained from the survey applied on a Likert scale.

## 4. Results

The results of the study are presented sequentially, commencing with a descriptive analysis of the characteristics of the respondents to identify the perceptions of those students and the SEM analysis to address the research objective.

The majority of the students surveyed are female (53%) and male (47%), and are enrolled in the following degree programmes: human medicine (18%), administration (16%), accounting (16%), and economics (15%).

**Figure 2.**
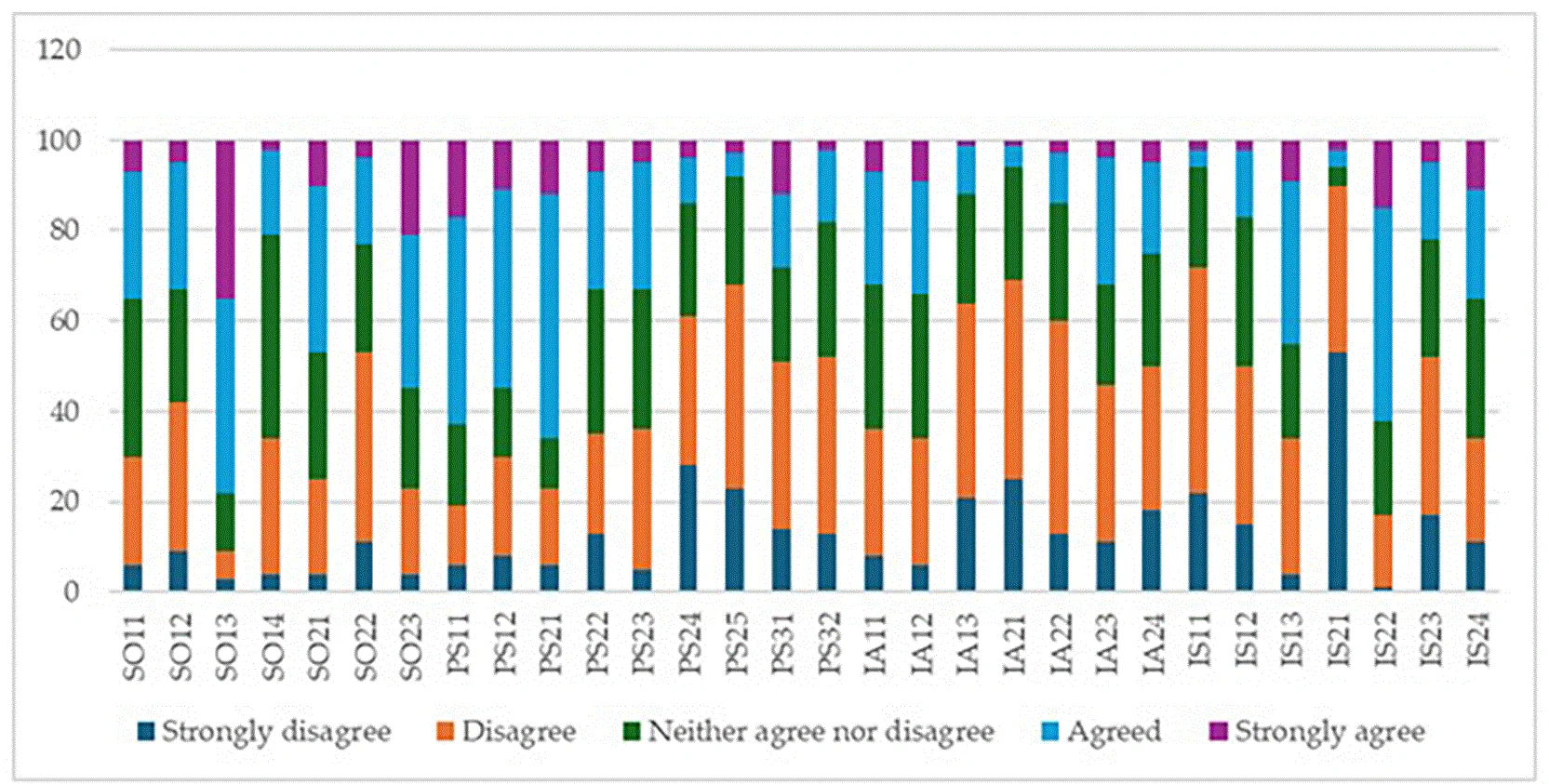
Description of perception. *Note:* Obtained from the survey applied on a Likert scale 1: strongly disagree; 2: disagree; 3: neutral; 4: agree; 5: strongly agree.

In relation to social factors, the majority of students expressed disagreement with the notion that family members exert influence over their decision-making processes (42%). Conversely, a significant proportion of students concurred with the assertion that members of their immediate social circle tend to offer mutual support (43%) and acknowledged the presence of considerable challenges within their family environment (37%) ( Figure 2).

With regard to the psychological factors under consideration, it is evident that the majority of students (46%) adopt a proactive stance towards their university studies, while a significant proportion (44%) express a strong motivation to persevere in their academic pursuits. Furthermore, there is a notable consensus among the student body that their approach to academic endeavours is consistently positive (54%).

With regard to the matter of academic integration, it is evident that students have expressed their disagreement with inflexible schedules (47%), as well as the notion that university teachers are more demanding than school teachers (44%), and the perception that the university environment is more arduous than the school environment (43%).

With regard to social integration, students indicate that they strongly disagree with the assertion that they encounter any difficulty in living alone (53%), and also with the assertion that their family has experienced any financial difficulties (50%). Furthermore, they express disagreement with the statements that they are not entirely certain about their chosen career (35%) and that they have experienced any health problems (35%) in Table 2.

**Table 2.** Characteristics of respondents.

Characteristics of respondents	Description	Frequency		%
**Gender**	**(1) Male, (2) Female**	**Male**	47	47
		**Female**	53	53
**Vocational school**	**Administration**	16		16.0
	**Biology**	1		1.0
	**Accounting**	16		16.0
	**Law**	7		7.0
	**Economics**	15		15.0
	**Nursing**	2		2.0
	**Biomedical Engineering**	2		2.0
	**Systems Engineering**	1		1.0
	**Electrical Engineering**	2		2.0
	**Electronic Engineering**	7		7.0
	**Human Medicine**	18		18.0
	**Veterinary Medicine**	7		7.0
	**Obstetrics**	2		2.0
	**Psychology**	4		4.0

Variables	Description		Perception (%)				
			Strongly disagree (1)	Disagree (2)	Neither agree nor disagree (3)	Agree (4)	Strongly agree (5)
**Social**	**SO1: Integration with the environment**	SO11: You find it easy to make friends in the university environment	6.0	24.0	**35.0**	28.0	7.0
		SO12: Easily integrates into any group in your university environment	9.0	**33.0**	25.0	28.0	5.0
		SO13: People in the environment tend to support each other	3.0	6.0	13.0	**43**	35.0
		SO14: The institution is concerned with promoting healthy coexistence in the environment	4.0	30.0	**45.0**	19	2.0
	**SO2: Family environment**	SO21: Your family environment has experienced serious difficulties	4.0	21.0	28.0	**37.0**	10.0
		SO22: Your family members influence your decisions	11.0	**42.0**	24.0	19.	4
		SO23: In your family, household chores are distributed	4.0	19.0	22.0	**34**	21.0
**Psychological**	**PS1: Previous behaviours**	PS11: Has a proactive attitude towards university studies	6.0	13.0	18.0	**46.0**	17.0
		PS12: Is motivated to continue their university career	8.0	22.0	15.0	**44.0**	11.0
	**PS2: Attitudes**	PS21: Their attitude towards their studies is always positive	6.0	17.0	11.0	**54.0**	12.0
		PS22: Emotional skills significantly influence their decisions	13.0	22.0	**32.0**	26.0	7
		PS23: Considers that their psychological health is in optimal condition	5.0	31.0	**31.0**	28.0	5.0
		PS24: Workshops are held on controlling psychological aspects related to stress.	28.0	**33.0**	25.0	10.0	4
		PS25: Considers that continuing a university career is important for their future life	23.0	**45.0**	24.0	5.0	3.0
	**PS3: Subjective standards**	PS31: Understands the importance of pursuing higher education	14.0	**37.0**	21.0	16.0	12
		PS32: Learn about the regulatory aspects of higher education teaching	13.0	**39.0**	30.0	16.0	2.0
**Academic integration**	**IA1: University characteristics**	IA11: Presents learning difficulties given that the Higher Education Institution faces problems in applying teaching strategies	8.0	28.0	**32.0**	25.0	7.0
		IA12: Does not have an organisational role in their studies	6.0	28.0	**32.0**	25.0	9.0
		IA13: Perceives that university is more difficult than school	21.0	**43.0**	24.0	11.0	1.
	**IA2: Institutional characteristics**	IA21: Considers that university lecturers are more demanding than school teachers	25.0	**44.0**	25.0	5.0	1
		IA22: Has inflexible schedules compared to those of the educational institution	13.0	**47.**	26.0	11.0	3
		IA23: Has had problems with the documentation requested by the university institution	11.0	**35.0**	22.0	28.0	4.0
		IA24: Perceives that teachers have not met expectations in teaching their classes	18.0	**32.0**	25.0	20.0	5
**Social integration**	**IS1: Family characteristics**	IS11: Your family has experienced financial difficulties	22.0	**50.**	22.0	4	2
		IS12: Has had any health problems	15.0	**35.0**	33.0	15.0	2
		IS13: Has any family member had a health problem?	4.0	**30.0**	21.0	36.0	9.0
	**IS2: Individual characteristics**	IS21: Has some difficulty living alone	**53.0**	37.0	4.0	4.0	2.
		IS22: The time spent attending to each scholarship recipient or applicant is considered optimal.	1.0	16.0	21.0	**47.0**	15.0
		IS23: Not entirely sure about chosen career	17.0	**35**	26	17	5
		IS24: Is there any discrimination based on their habits and/or customs?	11.0	23.0	31.0	**24.0**	11.0

*Note:* Obtained from the survey applied on a Likert scale: 1: strongly disagree; 2: disagree; 3: neutral; 4: agree; 5: strongly agree.

The present study aims to evaluate the psychosocial factors that explain academic dropout among scholarship students at a public university. To this end, a Structural Equation Model (SEM) analysis will be performed in order to pay close attention to construct validity, reliability, and the testing of hypotheses that contribute to this research.

With regard to the individual reliability of the items, all weights are above 0.707, with the exception of fifteen items belonging to the social, psychological, academic integration, and social integration constructs. Nevertheless, these items were retained on the basis that their content is also important in defining each of the constructs. In addition, the individual reliability of the items is adequate, as demonstrated in Table 3. ^51^ Initially, an evaluation was conducted to ascertain the potential for multicollinearity between the constructs. ^52^

**Table 3.** Individual item reliability (Formative).

Latent variable	External weights	VIF
SO11	0.749	1.761
SO12	0.611	1.360
SO13	0.573	1.450
SO14	0.707	1.566
SO21	0.604	1.324
SO22	0.624	1.689
SO23	0.775	1.934
PS11	-0.091	1.806
PS12	0.337	1.448
PS21	-0.082	1.741
PS22	0.402	1,437
PS23	0.731	1.460
PS24	0.687	1.479
PS25	0.765	1.809
PS31	0.766	1.742
PS32	0.278	1.106
IA11	0.665	1.909
IA12	0.661	1.820
IA13	0.479	1.934
IA21	0.618	2.383
IA22	0.475	1.358
IA23	0.743	2.395
IA24	0.720	2.524
IS11	0.701	1.580
IS12	0.180	1.098
IS13	0.506	1.240
IS21	0.230	1.218
IS22	0.700	1.496
IS23	0.816	1.855
IS24	0.795	1.711

*Note.* Obtained from survey processing.

In each of the partial multiple regressions, the VIF index for each of the exogenous constructs is less than 5. Therefore, we can affirm that there are no multicollinearity problems between the exogenous constructs of each endogenous variable, as shown in Table 3.

With regard to the internal consistency of the measurement scales, it is evident from Table 4 that the composite reliabilities of the constructs are greater than 0.7, with the exception of the psychological constructs. This observation serves to affirm the importance of the items or manifest variables for the model. In addition, the elevated Cronbach's alpha values (IA: α = 0.757; IS: α = 0.682; PS: α = 0.616; SO: α = 0.789) substantiate the inherent consistency and reliability of the instruments.

**Table 4.** Composite reliability.

Construct	Composite reliability
Academic integration (AI)	0.819
Social integration (SI)	0.779
Psychological (PS)	0.690
Social (SO)	0.847

*Note:* Obtained from survey processing.

The AVE (Average Variance Extracted) coefficient represents the proportion of variance in the construct that can be explained by its indicators, and it has been verified that this coefficient is greater than 0.5 for all constructs, as shown in Table 5. We can therefore affirm that there is convergent validity for each of the latent variables. ^53^

**Table 5.** Average variance extracted.

Construct	Average extracted variance (AVE)
Academic integration (AI)	0.598
Social integration (SI)	0.574
Psychological (PS)	0.583
Social (SO)	0.545

*Note:* Obtained from survey processing.

Discriminant validity analysis between constructs by calculating HTMT coefficients ^54^ and using the criteria of. ^53^ Table 6 shows that all constructs achieve discriminant validity according to the Forner-Lacker criterion and the stricter HTMT criterion ^55^ therefore, all constructs measure different aspects.

**Table 6.** Discriminant validity.

Construct	Academic integration (AI)	Social integration (SI)	Psychological (PS)
Academic integration (AI)	-----	-----	-----
Social integration (SI)	0.957	-----	-----
Psychological (PS)	0.849	0.806	-----
Social (SO)	0.815	0.809	0.755

*Note:* Obtained from survey processing.

In order to analyse the structural model and conduct hypothesis testing, it is hypothesised that a relationship exists between the constructs. The findings indicate that social factors related to integration with the environment and the family environment do not provide a satisfactory explanation for academic dropout as measured by academic integration (β = 0.228, t = 0.534, p < 0.593). Similarly, these factors do not provide a satisfactory explanation for academic dropout measured by social integration (β = 0.227, t = 0.579, p < 0.563). Contrary to the psychological factors that have been measured in previous studies on behavioural and subjective norms, it has been demonstrated that attitudes and subjective norms are more effective in explaining academic dropout when measured by academic integration (β = 0.836, t = 2.009, p < 0.045) and social integration (β = 0.747, t = 1.909, p < 0.056) ( Table 7).

**Table 7.** Hypothesis testing.

Hypothesis	Effect	Path coefficients	t-value	p-value	Supported?
Social (SO) -> Academic integration (AI)	+	0.228	0.534	0.593	NO
Social (SO) -> Social integration (IS)	+	0.227	0.579	0.563	NO
Psychological (PS) -> Academic integration (AI)	+	0.836	2.009	0.045	YES
Psychological (PS) -> Social integration (SI)	+	0.747	1.909	0.056	YES

*Note:* Obtained from survey processing.

Conversely, the results of the SEM analysis are presented for each of the 30 items of the four constructs considered: social integration, psychological integration, academic integration, and social integration. The results indicate that four items pertaining to the psychological constructs PS11, PS21, and PS32, as well as the social integration item IS12, are non-significant ( Table 8).

**Table 8.** Results of the SEM statistical test.

Latent variable	Description	Effect	Weights	t-value	p-value
**Social**	SO11: It is easy for you to make friends in the university environment university environment	+	0.653	7.69	0.000
	SO12: Easily integrates into any group in your university environment	+	0.599	6,737	0.000
	SO13: People in the environment tend to support each other	+	0.479	4,763	0.000
	SO14: The institution is concerned with promoting healthy coexistence in the environment	+	0.69	7,303	0.000
	SO21: Has your family environment experienced any serious difficulties	+	0.589	5,461	0.000
	SO22: Your family members influence your decisions	+	0.449	4.121	0.000
	SO23: In your family, tasks are distributed in the household	+	0.654	8,058	0.000
**Psychological**	PS11: Has a proactive attitude towards their university studies	-	-0.064	0.494	0.621
	PS12: Is motivated to continue with their university studies at university	+	0.295	2.552	0.001
	PS21: Attitude towards studies is always positive	+	0.033	0.259	0.796
	PS22: Emotional skills significantly influence significantly on their decisions	+	0.385	3,586	0
	PS23: Considers that their psychological health is in optimal condition	+	0.658	7.822	0.000
	PS24: Workshops are held to control psychological aspects related to stress	+	0.635	9,341	0.000
	PS25: Do you consider that continuing a university degree is important for your future life	+	0.58	7,824	0.000
	PS31: Understand the importance of pursuing higher education higher education	+	0.65	8,768	0.000
	PS32: Learn about the regulatory aspects of higher education in higher education	+	0.193	1,484	0.138
**Academic integration**	AI11: Presents learning difficulties given that the Higher Education Institution faces problems in applying teaching strategies	+	0.678	9.33	0.000
	IA12: Does not have an organisational role in their studies	+	0.667	9.524	0
	IA13: Perceives that the university environment is more difficult than the school environment	+	0.285	2,233	0.026
	IA21: Considers that university teachers are more demanding than school teachers	+	0.376	3.857	0.000
	IA22: Has inflexible schedules unlike those of the educational institution	+	0.332	3.448	0.001
	IA23: Has presented problems with the documentation requested requested by the university institution	+	0.654	9.02	0.000
	IA24: Perceives that teachers have not met expectations in teaching their classes	+	0.648	7,869	0.000
**Social integration**	IS11: Has your family experienced any financial problems?	+	0.644	6.73	0.000
	IS12: Have you had any health problems?	+	0.134	0.927	0.354
	IS13: Has any family member had any health problems?	+	0.359	2.889	0.004
	IS21: You have some difficulties living alone	+	0.192	1,768	0.077
	IS22: The time spent attending to each scholarship recipient or applicant is considered optimal	+	0.644	7.544	0.000
	IS23: Not entirely sure about the chosen degree programme	+	0.677	9.929	0
	IS24: There is some discrimination against your habits and/or customs	+	0.755	14,052	0.000

*Note:* Obtained from the processing of the surveys.

**Figure 3.**
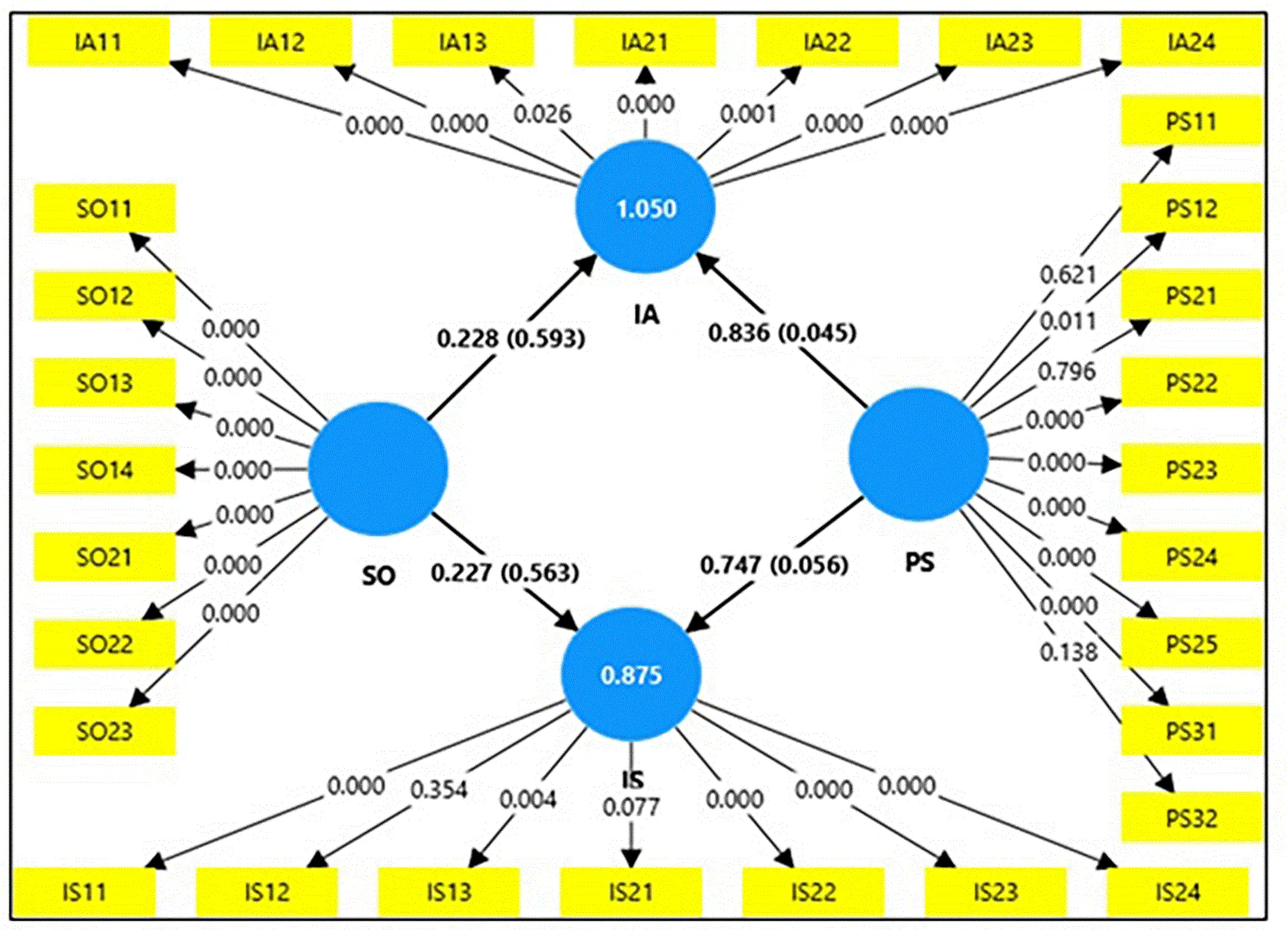
Model analysis using SEM. *Note:* Obtained from survey processing.

On the other hand, the structural equation model uses the results of confirmatory factor analysis, whose indices yield the following results: χ ^2^ = CMIN/df = 1.64, GFI = 0.923, AGFI = 0.955, NFI = 0.906, CFI = 0.996, RMSEA = 0.081, and SRMR = 0.100, demonstrating that the indices of the constructs in the estimated model are adequate to explain the social and psychological factors that account for academic dropout among scholarship students at a Peruvian public university ( Table 9 and Figure 3).

**Table 9.** Goodness-of-fit index results.

Model	CMIN/DF	GFI	AGFI	NFI	CFI	RMSEA	SRMR
Study model	1.649	0.923	0.955	0.906	0.996	0.08	0.100
Recommended value	Acceptable 1-4	>0.95	>0.9	>0.9	>0.9	<0.08	<0.100
Decision	Complies	Complies	Complies	Complies	Complies	Complies	Complies

*Note.* Obtained from survey processing.

## 5. Discussions

The findings of the present study demonstrate that psychosocial factors are a contributing element in the decision of scholarship students to withdraw from their academic studies. These results are in alignment with those of previous research conducted by. ^28, 35^ The present study identifies academic self-regulation, self-efficacy, and social support as key determinants of university persistence. These findings correspond with those of the aforementioned authors, who also identified the importance of social integration and the family environment in student retention. The present study's findings demonstrate a robust correlation between the capacity to establish amicable relationships and the capacity to coexist harmoniously with individuals who have chosen to discontinue their academic pursuits. This observation aligns with the identification of institutional commitment as a protective factor in the systematic review conducted by the aforementioned authors.

In addition, the results of the present study are consistent with those of the research conducted by Ramos ^32^ and Tlalajoe-Mokhatla. ^25^ These researchers demonstrated that low academic performance, workload, and negative perceptions of the family environment have a detrimental effect on student motivation and increase the risk of dropout. The findings of the present study demonstrate that the family environment is a significant predictor of student attrition, thus indicating that a paucity of support from family members can directly influence students' decisions to abandon their studies.

In contrast, the findings of Vega et al. and Vilchez ^31, 56^ serve to reinforce the evidence that psychological factors, including depressive symptoms, suicidal ideation, insomnia, and insufficient parental support, are associated with an elevated risk of academic disengagement. The present study has identified a correlation between psychological well-being and emotional skills on the one hand, and university retention on the other. This finding highlights the importance of psychosocial interventions within higher education institutions in order to mitigate the adverse effects identified.

Kocsis & Molnár ^57^ The psychological, realm our findings on mental health as a protective factor are in accordance with those of García et al. ^13^ who observe that “the pressure to maintain high grades in order to retain the scholarship generates chronic stress, affecting performance” (p. 12). Nonetheless, there is a divergence of opinion regarding the magnitude of the effect. Whilst the aforementioned study Garcia et al. ^13^ attributes greater weight to economic variables, the present study demonstrates that “knowledge of institutional norms” is the most influential predictor. This finding is in accordance with that of the study Kocsis and Molnár ^57^ conducted by the second author, who identified that clarity in academic expectations reduces uncertainty among scholarship students.

In contrast to the findings presented above, the study by Nuñez ^28^ emphasised the role of institutional and academic factors in dropout rates. This differs partially from the results obtained in this study, since the model developed herein highlighted the influence of subjective norms in the education system as the most influential factor. This finding suggests that enhancing comprehension and adaptability to institutional stipulations may substantially mitigate the inclination to withdraw among scholarship students.

In the Peruvian context, Salazar ^40^ dentified that factors such as rural origin and mother tongue have an impact on scholarship loss. This finding is at odds with our results, which indicate that the family environment, rather than demographic variables, is the critical factor. The observed discrepancy may be attributed to methodological disparities, given that Salazar ^40^ employed probit models, whereas our quantitative methodology placed greater emphasis on psychosocial constructs. Nevertheless, the consensus of both studies is that there is a requirement for comprehensive policies, as suggested by ^58^: “Scholarships must be complemented by mentoring and curricular flexibility to address multidimensional vulnerabilities” (p. 320).

It is therefore important for public universities to implement an initial risk-assessment process that combines available administrative information, such as prior academic performance, educational background, socioeconomic status, scholarship modality, change of residence, first-generation college student status, and self-reported support needs. Huynh-Cam et al. (2025) noted that demographic, family, and financial information available before the first semester should be used to identify students who require early assistance. ^59^ However, such risk classification should never be used to exclude, penalize, or withdraw scholarships; rather, it should trigger complementary institutional support.

Chen et al. (2024) identified absence, medical or personal leave, student loans, and required credits as relevant variables for the early detection of first-year dropout risk. ^60^ These findings support the view that public universities should not wait until final grades are released, because intervention at that stage may be too late to prevent the cumulative process of academic disengagement.

For their part, Mareş et al. (2026) argued that a holistic and coordinated approach is consistent with the emphasis on early detection, continuous monitoring, academic and psychosocial counseling, tutoring, flexible educational responses, and shared responsibility across institutions and individual support services. ^61^ Accordingly, the authors maintain that dropout prevention should integrate academic, psychological, social, financial, organizational, and cultural dimensions.

Finally, the work of Franz & Paetsch ^26^ on social integration in working students corroborates our observation that “those employees with strong university networks are less likely to drop out” (p. 5), although their study focuses on teaching careers, while the present study covers multiple disciplines. This convergence underscores the universality of social integration as a retention factor, irrespective of the academic context.

## 6. Conclusions

The study identified associations between social and psychological factors and academic and social integration among Beca 18 scholarship recipients with low academic performance at a Peruvian public university. Psychological factors showed the strongest relationship with academic integration; however, this finding does not represent a demonstrated effect on dropout because participant withdrawal or continued enrollment was not measured. Social relationships did not reach statistical significance, although their effect magnitudes were below the minimum detectable effect given the available sample. Consequently, the findings should be considered exploratory, limited to the institutional context analyzed, and subject to confirmation through longitudinal, multi-institutional studies with larger samples.

Theoretically, the present findings serve to reinforce the necessity of integrating psychological perspectives into models of university dropout, a requirement that is in alignment with studies emphasising self-regulation and mental health as pivotal factors in the retention of students. Methodologically, the use of structural equations enabled the unravelling of complex interactions between latent variables, thus validating the usefulness of quantitative approaches to analyse multi-causal phenomena. Nevertheless, the restricted impact of social elements may be indicative of deficiencies in the operationalisation of constructs such as family assistance or institutional coexistence, domains that necessitate further qualitative investigation.

Among the study's limitations, the exclusive focus on a single university and a small sample of scholarship recipients with low academic performance is particularly salient, thereby restricting the generalisation of results. Moreover, the exclusion of contextual variables, such as geographical origin or workloads, may have resulted in the omission of determining factors in contexts of economic vulnerability. It is recommended that future research endeavours encompass a more diverse sample and adopt a mixed methods approach to encompass the subjective dimensions that are not amenable to quantification through Likert scales.

The practical implications of this study indicate a necessity to formulate educational policies that incorporate ongoing psychological support, academic mentoring, and emotional skills training for scholarship recipients. The implementation of institutional adaptation programmes, with a focus on clarifying normative expectations and reducing academic anxiety, has the potential to mitigate the risk of dropout. These efforts should be complemented by strategies that foster institutional belonging, even if their direct impact was not statistically significant in this study.

In future research, it is recommended that the role of socioeconomic variables not previously considered be explored. Such variables may include family financial stability and access to technological resources, which could modulate the relationship between psychosocial factors and dropout. Conversely, longitudinal research would facilitate analysis of how these factors evolve throughout the university trajectory, thereby identifying critical windows for preventive interventions.

The aggregate contribution of this study is that it highlights that academic drop-out among scholarship students is a multifaceted phenomenon, with psychological components emerging as primary determinants. Whilst the findings challenge established socially-centred paradigms, they nevertheless pave the way for innovation in the field of evidence-based retention strategies. The combination of methodological rigour and sensitivity to student realities remains pivotal to the transformation of educational systems into environments that are genuinely inclusive.

## Limitations

The study has several limitations, beginning with the use of an academic average below 10 as a proxy variable to identify students in a situation of academic vulnerability. This is not a direct measure of dropout because the database used does not allow actual dropout to be identified or temporary interruption, institutional withdrawal, transfer, and permanent exit from the higher education system to be distinguished.

Another limitation concerns the cross-sectional design. Social, psychological, and integration factors were measured at a single point in time through self-report; therefore, temporal precedence could not be established, nor could it be verified that psychosocial conditions preceded subsequent changes in students’ academic trajectories. Consequently, the structural coefficients represent statistical associations within the sample rather than causal relationships.

Furthermore, because participants were drawn from a single public university, institutional characteristics, Beca 18 support mechanisms, academic demands, program offerings, and the territorial context may differ across universities.

Future research should therefore use administrative records on enrollment, withdrawal, and persistence; include additional universities and larger samples; and follow students for at least two academic periods.

## Ethics review

The study involved an anonymous online questionnaire administered via Google Forms (non-interventional; no clinical procedures or experimental manipulation; no directly identifiable data collected). This work originated as a formative assignment within a postgraduate research methods course at the Graduate School of the National University Pedro Ruiz Gallo (UNPRG), ^62^ and it was not submitted as a thesis- or degree-related protocol. Therefore, no formal ethics committee/IRB approval or waiver reference number is available. In the manuscript we now cite the relevant UNPRG institutional documents (Code of Ethics and Scientific Integrity for Research; Regulation of the Research Ethics Committee) and we attach an updated sworn statement explaining this context. The study also adhered to the UNPRG Code of Ethics and Scientific Integrity for Research (Resolution No. 1134-2018-R).

## Informed consent

Informed consent was obtained in written electronic form (e-consent). The consent statement was displayed on the first page of the Google Form, and respondents could proceed only after actively selecting the agreement option (“I agree”). This has been clarified in the manuscript, and an updated e-consent form is included as a supplementary file.

## Data Availability

Zenodo. Psychosocial factors explaining academic dropout among scholarship students at a public university in Perú, https://doi.org/10.5281/zenodo.17735445 ^63^ This project contains the following underlying data:
• Database_ Information from data collected. • Estimated results. • Informed consent. • Sworn statement. Data is available under Creative Commons Zero v1.0 Universal This project contains the following extended data: Zenodo. Psychosocial factors explaining academic dropout among scholarship students at a public university in Perú, https://doi.org/10.5281/zenodo.17735445 ^63^
•
Figure 1. Theoretical design obtained from university dropout models that include social and psychological factors. •
Figure 2. Description of perception. •
Figure 3. Model analysis using SEM. Data is available under Creative Commons Zero v1.0 Universal
