# Supplementary material for: Psychosocial Factors Explaining Academic Dropout among Scholarship Students at a Public University in Peru
Source: F1000Res. 2026 Aug 21;15:405. Originally published 2026 Mar 17. [Version 2] doi: 10.12688/f1000research.174541.2 (PMC13562923; doi:10.12688/f1000research.174541.2)
Supplement: Peer review history

# Reviewer report: Reviewer response for version 2

**DOI:** 10.5256/f1000research.208206.r513406

**Published:** 2026-09-09

**Authors:** Thao-Trang Huynh-Cam

**Affiliations:** 1 Dong Thap University, Cao Lanh, Vietnam

Congratulations. The current version of the manuscript has accordingly been revised.  I accept this current version for indexing.

Is the work clearly and accurately presented and does it cite the current literature?

Yes

If applicable, is the statistical analysis and its interpretation appropriate?

Yes

Are all the source data underlying the results available to ensure full reproducibility?

Yes

Is the study design appropriate and is the work technically sound?

Yes

Are the conclusions drawn adequately supported by the results?

Yes

Are sufficient details of methods and analysis provided to allow replication by others?

Yes

Reviewer Expertise:

E-learning, Information management in education, Teaching English as a Second Language, Education management, Educational data mining, Decision making in education, ICT in learning and teaching foreign languages, English Linguistics

I confirm that I have read this submission and believe that I have an appropriate level of expertise to confirm that it is of an acceptable scientific standard.

# Reviewer report: Reviewer response for version 2

**DOI:** 10.5256/f1000research.208206.r513405

**Published:** 2026-08-22

**Authors:** Marco Agustin Arbulu Ballesteros

**Affiliations:** 1 Universidad Cesar Vallejo, Trujillo, Peru

I thank the authors for their careful revision of the manuscript. The changes introduced in this version adequately address the comments and recommendations raised in my previous review, and I have no further comments to make. I am happy to approve the revised version of this article.

Is the work clearly and accurately presented and does it cite the current literature?

Yes

If applicable, is the statistical analysis and its interpretation appropriate?

Yes

Are all the source data underlying the results available to ensure full reproducibility?

Yes

Is the study design appropriate and is the work technically sound?

Yes

Are the conclusions drawn adequately supported by the results?

Partly

Are sufficient details of methods and analysis provided to allow replication by others?

Partly

Reviewer Expertise:

Multivariate modelling based on SEMModelamiento multivariado basado en SEM

I confirm that I have read this submission and believe that I have an appropriate level of expertise to confirm that it is of an acceptable scientific standard.

# Reviewer report: Reviewer response for version 1

**DOI:** 10.5256/f1000research.192452.r490801

**Published:** 2026-09-07

**Authors:** Rosmery Sabina Pozo Enciso

**Affiliations:** 1 Universidad Nacional Micaela Bastidas de Apurimac, Abancay, Apurimac, Peru

The manuscript entitled “Psychosocial factors explaining academic dropout among scholarship students at a public university in Peru” addresses an important issue for university management and student-retention policies. The study seeks to identify the psychosocial factors associated with academic dropout among students who received Beca 18 scholarships in 2024. It uses a quantitative approach, a descriptive-explanatory design, and structural equation modeling.

 INTRODUCTION

 Overall, the article addresses a relevant and timely topic of interest to researchers, university authorities, and administrators of public scholarship programs. Its principal contribution lies in jointly examining social, psychological, academic, and institutional dimensions, thereby demonstrating that discontinuing one’s studies is a multidimensional phenomenon rather than an exclusively economic one. Furthermore, the inclusion of students from different degree programs strengthens the descriptive scope of the study.

 METHODOLOGY

 The manuscript is appropriately organized and presents a coherent progression from the research problem and theoretical framework to the methodology, results, and conclusions. Of particular value is the use of structural equation modeling to analyze relationships among latent variables, together with the reporting of reliability, convergent validity, discriminant validity, and model-fit indicators. The findings highlight the importance of attitudes, motivation, psychological well-being, and knowledge of institutional regulations in scholarship students’ academic and social integration.

 RESULTS

 It is also commendable that the study acknowledges its limitations, particularly the use of a relatively small sample, its focus on a single university, and the need to incorporate contextual and socioeconomic variables in future research. This acknowledgment supports a cautious interpretation of the findings and appropriately delineates the limits of their generalizability.

 The practical implications are relevant because the manuscript proposes ongoing psychological support, academic mentoring, the strengthening of emotional skills, and institutional adaptation programs to reduce academic anxiety and promote scholarship students’ continued enrollment. In addition, making the dataset, estimated results, and consent documents available in an open repository enhances the transparency and reproducibility of the research.

 RECOMMENDATIONS

 The following recommendations are offered to strengthen the final version of the article:

 Expand the justification for the measurement model.

 The authors should explain more precisely why the constructs were modeled using formative indicators and demonstrate that this specification is consistent with their theoretical definitions. Doing so will facilitate an appropriate understanding of the outer weights, reliability, average variance extracted (AVE), and discriminant validity.

 Strengthen the discussion of practical implications.

 The Discussion should translate the findings into specific measures for public universities and Beca 18 program administrators. Potential measures include early-warning systems, differentiated tutoring, guidance on academic regulations, periodic assessments of psychological well-being, and referral protocols for specialized services.

 FINAL RECOMMENDATION FOR ACCEPTANCE

 After reviewing the manuscript, I consider the study to make a relevant contribution to understanding the factors associated with dropout risk among scholarship students in Peruvian higher education. The research has an adequate theoretical foundation, reports findings that are useful for university management, and offers practical implications for providing psychological, academic, and institutional support to a vulnerable student population.

 The observations raised do not invalidate the study’s central contribution; however, addressing them would improve the article’s statistical precision, methodological coherence, and interpretive caution. I therefore recommend accepting the manuscript subject to revisions, given its academic relevance, practical usefulness, and potential contribution to the design of preventive student-retention strategies at public universities in Peru.

Is the work clearly and accurately presented and does it cite the current literature?

Yes

If applicable, is the statistical analysis and its interpretation appropriate?

Yes

Are all the source data underlying the results available to ensure full reproducibility?

Yes

Is the study design appropriate and is the work technically sound?

Yes

Are the conclusions drawn adequately supported by the results?

Yes

Are sufficient details of methods and analysis provided to allow replication by others?

Yes

Reviewer Expertise:

PhD in Education, with Master’s degrees in Foreign Language Teaching and Social Management, a Bachelor’s degree in Education and a law degree.

I confirm that I have read this submission and believe that I have an appropriate level of expertise to confirm that it is of an acceptable scientific standard.

# Reviewer report: Reviewer response for version 1

**DOI:** 10.5256/f1000research.192452.r482065

**Published:** 2026-06-04

**Authors:** Thao-Trang Huynh-Cam

**Affiliations:** 1 Dong Thap University, Cao Lanh, Vietnam

This study aims to provide a practical insight to factors influencing scholarship students, having the low academic performance in 18 programs at a Peruvian public university in 2024. The ideas in the manuscript are highly robust, very practical, sound, and interesting. This paper fulfils the crucial gaps in practices and literature regarding scholarships students’ withdrawals or underperformances. The study is well-conceived, methodologically sound, and addresses a distinct gap in the literature and practice. This study has potentials to contribute to the theories and practices on student dropouts. However, in order to enhance the impact, I have following suggestions.   

1. Discussion section should provide practical suggestions to policymakers of public universities in Peru so that they have enough time to implement proactive preventive measures.  
2. References: Please cite at least one reference from the journal F1000Research.  
3. Below are several published papers for your references.
  1. Huynh-Cam, T. T., Chen, L. S., & Lu, T. C. (2025). Early prediction models and crucial factor extraction for first-year undergraduate student dropouts. *Journal of Applied Research in Higher Education*, *17*(2), 624-639.
  2. Chen, L. S., Huynh-Cam, T. T., Nalluri, V., Lu, T. C., & Agrawal, S. (2024, September). Determining important features for first-year student dropouts using artificial intelligence algorithms. In *2024 6th International Workshop on Artificial Intelligence and Education (WAIE)* (pp. 128-133)
  3. Quincho-Apumayta, R., Carrillo Cayllahua, J., Ccencho Pari, A., Inga Choque, V., & Cárdenas-Valverde, J. (2025). University Dropout Among Indigenous University Students: A Global Systematic Review. *F1000Research*, *14*, 641.
  4. Mareș, G., Cârtiță-Buzoianu, C., Cojocariu, V. M., & Amălăncei, B. M. (2026). Policy Frameworks and Strategic Approaches for Preventing and Reducing University Dropout Rates. *Revista Romaneasca pentru Educatie Multidimensionala*, *18*(1), 14-28.

Is the work clearly and accurately presented and does it cite the current literature?

Yes

If applicable, is the statistical analysis and its interpretation appropriate?

Yes

Are all the source data underlying the results available to ensure full reproducibility?

Yes

Is the study design appropriate and is the work technically sound?

Yes

Are the conclusions drawn adequately supported by the results?

Yes

Are sufficient details of methods and analysis provided to allow replication by others?

Yes

Reviewer Expertise:

E-learning, Information management in education, Teaching English as a Second Language, Education management, Educational data mining, Decision making in education, ICT in learning and teaching foreign languages, English Linguistics

I confirm that I have read this submission and believe that I have an appropriate level of expertise to confirm that it is of an acceptable scientific standard, however I have significant reservations, as outlined above.

## References

[1] Huynh-Cam, T. T., Chen, L. S., & Lu, T. C. (2025). Early prediction models and crucial factor extraction for first-year undergraduate student dropouts. Journal of Applied Research in Higher Education, 17(2), 624-639.

[2] Chen, L. S., Huynh-Cam, T. T., Nalluri, V., Lu, T. C., & Agrawal, S. (2024, September). Determining important features for first-year student dropouts using artificial intelligence algorithms. In 2024 6th International Workshop on Artificial Intelligence and Education (WAIE) (pp. 128-133).

[3] : University Dropout Among Indigenous University Students: A Global Systematic Review. *F1000Research* .2025;14: 10.12688/f1000research.162508.2 10.12688/f1000research.162508.2 40657179

[4] Mareș, G., Cârtiță-Buzoianu, C., Cojocariu, V. M., & Amălăncei, B. M. (2026). Policy Frameworks and Strategic Approaches for Preventing and Reducing University Dropout Rates. Revista Romaneasca pentru Educatie Multidimensionala, 18(1), 14-28.

# Reviewer report: Reviewer response for version 1

**DOI:** 10.5256/f1000research.192452.r470465

**Published:** 2026-04-23

**Authors:** Marco Agustin Arbulu Ballesteros

**Affiliations:** 1 Universidad Cesar Vallejo, Trujillo, Peru

Here are the four most critical observations from a peer reviewer's perspective:

**1. Inappropriate use of PLS-SEM with formative indicators for theoretically reflective constructs**

 The model reports outer weights and VIF values under formative logic, but all four core constructs social, psychological, academic integration, and social integration — are conceptually reflective: the items are supposed to *reflect* an underlying latent trait, not *cause* it. Applying a formative specification to reflective constructs without explicit theoretical justification invalidates the interpretation of weights, AVE indices, and discriminant validity criteria (Fornell-Larcker, HTMT). The authors must either provide a rigorous theoretical rationale for the formative choice or reconceptualize the measurement model accordingly.

**2. Serious numerical inconsistencies between the abstract and the body of the article**

 The abstract reports GFI = 0.923, AGFI = 0.855, NFI = 0.90, and CFI = 0.896, while Table 9 shows AGFI = 0.955, NFI = 0.906, and CFI = 0.996. These are not minor rounding differences — CFI jumps from 0.896 to 0.996, a gap that separates a marginally acceptable fit from a near-perfect one. If the model was re-estimated between drafting the abstract and the final version, those changes needed to be documented. If these are typographical errors, they undermine confidence in every other reported figure. The manuscript must reconcile all fit statistics against the original SmartPLS or AMOS output files before resubmission.

**3. Absence of formal ethics approval and insufficient justification for its omission**

 The authors argue that the survey required no IRB approval because it originated as a postgraduate course activity. However, the instrument was administered to Beca 18 recipients with low academic performance a vulnerable population and collected data on sensitive psychological dimensions including mental health, discrimination, and family hardship. The "formative assignment" exemption does not extend to research conducted with publication intent. F1000Research requires ethics documentation for all human subjects research, and without a committee reference number the manuscript fails to meet the journal's own stated policies as well as international research ethics standards.

**4. Small single-site sample and questionable operationalization of the dependent variable**

 The study works with 100 students from a single Peruvian public university, treating low academic performance (GPA below 10) as a proxy for dropout. This decision is both theoretically and empirically problematic: a student with poor grades is not equivalent to a dropout, and the structural model estimates relationships among psychosocial constructs without ever directly measuring academic withdrawal. On top of that, the sample size is insufficient for a SEM model with 30 indicators and four latent constructs  the widely accepted rule of thumb calls for at least 10 observations per free estimated parameter. Together, these two issues make the reported path coefficients (particularly β = 0.836 for PS → AI) unstable and essentially ungeneralizable beyond this specific sample.

Is the work clearly and accurately presented and does it cite the current literature?

Yes

If applicable, is the statistical analysis and its interpretation appropriate?

Yes

Are all the source data underlying the results available to ensure full reproducibility?

Yes

Is the study design appropriate and is the work technically sound?

Yes

Are the conclusions drawn adequately supported by the results?

Partly

Are sufficient details of methods and analysis provided to allow replication by others?

Partly

Reviewer Expertise:

Multivariate modelling based on SEMModelamiento multivariado basado en SEM

I confirm that I have read this submission and believe that I have an appropriate level of expertise to confirm that it is of an acceptable scientific standard, however I have significant reservations, as outlined above.
